# An Investigation of the Practices of Australian Adults Experiencing Pain and Their Views of Australian Community Pharmacy Pain Management Services

**DOI:** 10.3390/pharmacy8040187

**Published:** 2020-10-13

**Authors:** John Mishriky, Ieva Stupans, Vincent Chan

**Affiliations:** Pharmacy, School of Health and Biomedical Sciences, RMIT University, Bundoora, VIC 3083, Australia; john.mishriky@rmit.edu.au (J.M.); ieva.stupans@rmit.edu.au (I.S.)

**Keywords:** pain management, chronic pain, quality of life, paracetamol, non-steroidal anti-inflammatory agents, drug therapy, pharmacies, pharmacists, community pharmacy services, Australia

## Abstract

Pain is a common and debilitating condition requiring appropriate assessment and management. The consequences of inadequate treatment of pain is well known; however, research investigating pain management practices and the benefits Australian community pharmacies offer in pain management are more limited. This study investigated the knowledge and practices of Australian adults experiencing pain, and their views of community pharmacy pain management services. A cross-sectional study was conducted using a pre-tested anonymous self-administered questionnaire. Participants were recruited from ten community pharmacies across metropolitan Melbourne, Australia. A total of 120 participants completed the questionnaire. Most reported that their pain interfered with their quality/functionality-of-life. Paracetamol was the pharmacological preference irrespective of pain severity experienced. Approximately 30% did not believe that visiting a community pharmacy is helpful in pain management, but many reported their pain management knowledge could be improved, yet more than 60% disagreed when asked whether they would rather visit a supermarket than their pharmacy to purchase analgesics. More than half believed that community pharmacies can and should offer more pain management services. This suggests that enhancing the involvement of community pharmacists can help bridge gaps in pain management knowledge, which may provide greater positive outcomes for patients experiencing pain symptoms.

## 1. Introduction

According to the International Association for the Study of Pain (IASP), pain is defined as an unpleasant sensory and emotional experience associated with actual or potential tissue damage [1,2]. It is a commonly presenting complaint seen in the Australian healthcare setting [3,4,5]. An estimated 20 per cent of Australian adults suffer from chronic pain, defined as pain that has been present for at least 3 to 6 months [2,6]. Research investigating the severity and impact pain symptoms have on the quality and functionality of sufferers is well documented. However, studies specifically investigating the views and practices of adults experiencing pain are variable and limited, particularly in the Australian community setting. Assessing the various pharmacological and non-pharmacological treatment strategies utilised by those experiencing pain can provide a basis for tailoring specific pain management services that meet patient needs. Additionally, exploring the views and needs of Australian adults experiencing pain can facilitate the structuring of pharmacy-specific pain management intervention programmes that can be implemented in Australian community pharmacy settings.

Australian community pharmacies are considered to be important sources of a wide range of healthcare services and are regularly the first point of contact for many patients due to convenience, accessibility and availability of many Over-The-Counter medications (OTCs) at reasonable costs [7,8,9]. Furthermore, patients frequently engage with pharmacists for pain treatment and patients frequently use OTCs to self-manage their pain [10]. Primary care management should be holistic and evidence-based (where possible) and incorporates both pharmacological and non-pharmacological approaches such as complementary therapies and comprehensive pain-management programmes [11]. Research investigating pain management practices and the potential benefits Australian community pharmacies offer in pain management is worth exploring. Pharmacists offer numerous services beyond the supply and counselling of medicines. In the area of pain management, pharmacists offer detailed analgesic medication reviews that are aimed to identify and address issues with medication safety and adherence. As an additional outcome of the medication review service, pharmacists are ideally placed to refer their patients to relevant health professionals if need be. It is therefore necessary to better understand how patients handle their pain symptoms, and what pharmacy-specific services they incorporate into their pain management therapy. Thus, given the currently limited published literature in this context, the aims of this study were to investigate the current views and practices of patients experiencing pain in the Australian community, and to investigate patient perspectives on the community pharmacy/pharmacist services currently offered for pain management, and thus, identify any potential opportunities for improvement in this space.

## 2. Materials and Methods

### 2.1. Ethics Approval

This study was approved by the Human Research Ethics Committee of RMIT University (Approval number: SEHAPP 63-18).

### 2.2. Study Participants

This study was a descriptive cross-sectional exploratory study designed to capture the current views and practices of Australian adults who experience pain. Data collection was conducted over an approximately 12-week period (January–March 2019). Participation involved completing an anonymous survey either on-line (via Qualtrics that was accessible using a provided computer tablet device) or by filling out a paper version of the survey. Completion of the survey took approximately 5–10 min and was open to English speaking adults who suffer from pain of different aetiologies. Participants were identified and recruited at 10 community pharmacies across a broad geographical area in metropolitan Melbourne in the state of Victoria, Australia. Recruitment of participants was conducted through convenience sampling by the community pharmacy staff members for Australian adults who visit a community pharmacy and request/purchase OTC and/or prescription analgesics for themselves, or those who request to talk to a community pharmacy staff member about their pain. The investigators were responsible for the initial pharmacy approvals obtained from the various Victorian community pharmacies.

### 2.3. Questionnaire

A structured self-administered anonymous survey was developed to collect a broad range of data from Australian adults suffering from pain (File S1). Qualtrics software was used to develop and deliver the on-line version of this survey.

Questions were developed under six sections. To ensure that a broad range of participant responses were captured, demographic data such as age, gender, employment status, tertiary qualifications, medical conditions present, allergies/sensitivities present and alcohol and smoking statuses were obtained. The next two sections of the survey consisted of questions relating to the participant’s pain status, which included questions on how long pain symptoms have been present for, and what the original cause of the main type of pain is (if known). Participants were asked to list all of their analgesic medications used and to rate how effective the analgesic medications were at relieving their symptoms. Additionally, respondents were asked to rate their level of pain and how their pain symptoms have impacted their quality and functionality of life on numerical scales.

To assess their knowledge and views, in the main body of the questionnaire, participants were presented with a number of pharmacological and non-pharmacological treatment options and were asked to select what treatment options they would use based on the severity of pain symptoms experienced (mild, moderate or severe pain).

The final section of the questionnaire consisted of a series of categorical questions (five-point Likert scale ranging from Strongly disagree to Strongly agree) investigating the participants’ views on their pain management strategies, whether their pain management strategies could be improved, and their views on the pain management programmes currently offered by community pharmacists at Australian community pharmacies.

The questionnaire underwent a series of pilot tests with a small group of pharmacists, pharmacy academics and chronic pain patients before final release. The survey was preliminary pre-tested for ease of use and validity, and to identify any technical or interpretative issues, and a second round of pilot tests were conducted before the questionnaire was made available to Australian adults who experience pain.

### 2.4. Data Analysis

Statistical tests and descriptive statistics were conducted (using the statistical software package SPSS version 25) to assess responses to the questionnaire. Microsoft Office software was used to collate the results and construct the tables included in this manuscript.

## 3. Results

Of the total of 134 participants who attempted the survey, 14 incomplete submissions were excluded, with 120 completed responses used for this study. Table 1 describes the participant demographics.

Around two-thirds of participants reported suffering from additional medical conditions. The most common comorbidities listed were hypertension, hypercholesterolaemia, arthritis and mental health disorders such as depression and anxiety. Occasional smokers were defined as those who do not smoke cigarettes on a daily basis, whereas regular smokers were defined as those who smoke any number of cigarettes daily. Additionally, occasional drinkers of alcohol were those who drank no more than 1–2 standard drinks some days per week or less, and regular drinkers were those who drank at least 1–2 standard drinks most days per week or more. Of the participants who smoke cigarettes, only 3% stated that they smoke to alleviate their pain. Additionally, close to 8% of participants also reported that they drink alcohol to help ease their pain symptoms.

Most participants who completed the survey fall into the category of experiencing pain that is ‘chronic’ in nature; more than 75% reported that they have been experiencing pain symptoms for greater than 12 months (Table 2). Approximately one-third of participants mentioned that they are unaware of the original cause of their main type of pain (Table 2).

Pain scores reported on the numerical pain scale were variable; however, nearly half the number of participants scored an 8 or above when asked to rate their highest level of pain experienced in the previous week (Table 3). When asked to report on how their pain interferes with their quality and functionality of life (Table 3), more than 25% of participants scored at least 8 in all of the quality/functionality of life parameters (with the exception of ‘movement’ and ‘appetite’).

Of the pharmacological preferences listed, paracetamol was the drug choice of participants to treat pain symptoms of all severities (mild, moderate and severe) followed by ibuprofen (Table 4).

There was a degree of uncertainty reported by participants, particularly when asked whether visiting a community pharmacy and collaborating with their community pharmacist is helpful in the context of pain management. Although the majority agreed that their pain management strategies could be improved, approximately one-third neither agreed nor disagreed that there are avenues for community pharmacies to improve and offer more pain management services (Table 5). Incidentally, more than 60% of respondents also disagreed/strongly disagreed when asked whether they would rather visit a supermarket than a community pharmacy to purchase their analgesics (Table 5).

## 4. Discussion

### 4.1. The Consequences of Inadequate Diagnosis and Treatment of Pain

This study examined the self-reported practices and opinions of Australian adults who experience pain of different aetiologies. Although most participants reported a strong belief in their knowledge of pain management, the results demonstrated that there was some degree of variability in the approach to managing pain symptoms of different severities.

Adults who experience symptoms of chronic pain most often seek assistance from multiple primary as well as allied health providers for pain relief [12]. From a therapeutic perspective, it is suggested that pain collectively is either undertreated, or mistreated altogether [13]. Inadequate diagnosis and treatment give rise to clinical as well as practical health concerns for those experiencing pain. Failure to adequately manage pain in general results in significant costs to the healthcare system as well as posing financial strain on sufferers and their families in dealing with pain management [9,12].

It is well established that symptoms of ongoing and persistent pain can directly impact other components of an individual’s physical and mental state due to the life changes that may develop as a consequence of living with chronic pain. Additionally, inadequate diagnosis and treatment of pain symptoms may catalyse the deterioration of a person’s mental health [14,15,16,17]. Numerous studies have identified links between inadequate pain treatment and a decline in psychological health regardless of the origin of pain [18]. For instance, in one sleep study, it was found that the polygraphic sleep recordings of chronic pain participants led to the recognition of specific patterns of disturbed sleep, and in severe cases, insomnia [19]. Another study also verified that chronic pain patients commonly experience sleep disturbances due to inadequate management of their pain [17]. The findings from this study also support this notion; more than 70% of respondents scored a 5 or more when asked to report how their pain interferes with their sleep. Additionally, research in this area confirms links between chronic pain and the potentiation of mental health conditions, such as anxiety and depression [18]. Although they were not specifically asked to comment on their mental health in this study, it is interesting to note that more than half the number of participants scored a 5 or higher when asked to rate how their pain affects their ‘enjoyment of life’. Failure to address the psychological impact, coupled with ongoing inadequate treatment of pain only serves to facilitate a downwards spiral in the quality and functionality of life of patients [13,20]. It is therefore imperative that effective pain management should be holistic, individualistic and readily available to patients, while also giving consideration to the connection chronic pain has to the physical and psychological components of health.

### 4.2. Evidence-Based Approaches to Pain

In this study, participants were presented with a series of case scenario questions and were asked to select their preferred treatment option depending on the severity of pain experienced. The simple analgesic paracetamol was the most commonly selected treatment option across all three pain severity levels (mild, moderate and severe) followed by ibuprofen. The mechanism of action of paracetamol is complex and not fully understood; however, it is suggested that paracetamol relieves pain through the prostaglandin inhibition pathway [21,22]. In Australia, paracetamol is available in many formulations and in different strengths, often combined with other ingredients such as caffeine, ibuprofen or codeine, with the latter available only with a prescription. The role of paracetamol as a noteworthy contender in the management of pain has always been a subject of debate. However, most up-to-date clinical resources indicate that paracetamol no longer holds a position in alleviating chronic pain especially in patients who experience chronic low back pain (which incidentally appears to be the most commonly selected pain experienced by participants of this study) [23,24,25].

Despite the lack of evidence supporting its use for some types of pain, paracetamol remained the most commonly selected choice compared to other clinically superior analgesics such as Non-Steroidal Anti-Inflammatories (NSAIDs) like ibuprofen and diclofenac. Research investigating the analgesic potential of paracetamol versus NSAIDs has been well documented. An excellent meta-analysis assessing the efficacy of paracetamol versus NSAIDs across different pain conditions concluded that ibuprofen was the far superior drug producing significantly better outcomes for patients experiencing acute pain, migraine pain and osteoarthritis pain [26]. Although paracetamol does not hold the same analgesic potential as NSAIDs, it is still regarded as a safer long-term option particularly in patients experiencing long-term pain [21]. Clinical resources also emphasise that while NSAIDs provide more positive health outcomes, they should only be used in patients with no contraindications and for short term treatment only [4,23,24]. Clinical recommendations also emphasise that NSAIDs are to be used with caution in patients with chronic pain given that symptoms are ongoing and persistent [23]. Although the rationale of why paracetamol was favoured was not specifically investigated in this study, these could be the reasons as to why participants preferred paracetamol over NSAIDs. Despite paracetamol’s favourability amongst participants, close to one-quarter reported that they do not believe that their analgesic medicines are providing adequate pain relief, even though more than half reported that they take their analgesics regularly. Outcomes reported in this section emphasise the important need for Australian adults experiencing pain to seek the expertise of qualified health professionals, such as pharmacists, who are equipped with the knowledge and tools to identify any potential inadequacy in treatment and offer alternative and more effective pain management strategies to their patients.

### 4.3. The Position of the Community Pharmacist in Pain Management

Chronic pain is a complex and often compounded condition that negatively impacts the quality and functionality of life of sufferers [27]. The results from the 2020 Australian National pain survey indicate that participants feel isolated particularly when dealing with health professionals who downgrade the complexity of managing their symptoms [28]. In one Australian study published in 2019, the authors identified that although participants reported an appreciation of the pharmacological counselling provided by pharmacists, some reported that pharmacists lacked empathy and listening skills [29]. This was also reflected by respondents of the national pain survey, which highlighted the necessity for health professionals to listen to the needs of their patients in private and without generalisation and judgement [28]. This has always been a cause for concern in community pharmacy settings where patients are usually consulted by pharmacists in the communal area of the pharmacy, thus jeopardising the privacy of the patient [30,31]. Although more than 50% of participants believed that Australian community pharmacies are beneficial and community pharmacists can and should offer more pain management services, around one-third neither agreed nor disagreed with this statement, suggesting a possible degree of unawareness from the consumers’ perspectives of exactly what pharmacists can provide beyond the dispensing of prescription medicines. Australian pharmacies are frequently visited by the community, and as such, pharmacists have an ideal opportunity to interact with and provide a safe environment for their patients when assessing their pain management needs [5,7].

Pharmacists are considered trust-worthy purveyors of healthcare who can translate their knowledge into constructive and adaptable practices tailored to their patients, especially those experiencing chronic pain [8,9]. Australian clinical resources recommend that chronic pain management should incorporate physical, psychological and pharmacological therapy in order to achieve positive and sustainable health outcomes [13]. Pharmacists in this area can expand on their engagement with patients by advising them of these recommendations and liaising with medical and allied health professionals to achieve better pain management outcomes for their patients. Due to their extensive pharmacology education, pharmacists are also ideally placed to identify medication interactions that may cause harm. This responsibility is further heightened in the context of chronic pain management; chronic pain often requires a multiplicity of analgesic medications in order to achieve sustainable and adequate pain relief [9]. The results from this study also reflect this; most participants listed numerous analgesic medicines they regularly use as part of their pain management approach. In circumstances where interactions have been identified, pharmacists then have an opportunity to bring this to the attention of their patients and thereby provide education on the quality and safe use of medicines. An excellent example of this was identified in an Australian study, where results showed that Australian community pharmacists were ideally placed to identify interactions between OTC analgesics and concomitant use of the anticoagulant warfarin, and all pharmacists involved recommended alternative OTC analgesics with less likelihood of causing adverse effects [32]. In the context of pain management, pharmacists also have an opportunity to encourage the trial of alternative pain management strategies that are consistent with the recommendations outlined in clinical therapeutic guidelines. However, a dilemma that Australian pharmacists and patients are faced with is the limited variability of analgesics available in community pharmacies. One study conducted recently in Australia reported that the only available pain-relieving medicines in community pharmacies (without a prescription from an authorised prescriber) are simple analgesics such as paracetamol and limited quantities of the NSAIDs aspirin, ibuprofen, diclofenac, naproxen and mefenamic acid [33]. Prior to its up-scheduling to ‘prescription-only’ in 2018, the mild opioid codeine was once available without a prescription in Australian community pharmacies in fixed low dose preparations with either paracetamol, ibuprofen or aspirin. Although the majority of community pharmacists in this 2019 study supported the up-scheduling, some reported that the intention of mitigating the misuse of codeine was not met, and only served as a barrier to patients requiring adequate pain relief in urgent situations [33].

Despite the limited pharmacological options available in Australian community pharmacies, more than half the number of respondents either disagreed or strongly disagreed when asked whether they would rather visit a supermarket than a pharmacy to purchase analgesics. It appears that many still value the importance of interacting with their pharmacists in the context of pain management and, as such, community pharmacists have a vast opportunity to expand on their recognition and engagement with patients and offer more than dispensing and supply of prescription medicines. Additionally, the results from the national pain survey also support this where participants suggested the need for community pharmacies to advertise their pain management services given that participants reported being unaware of what else pharmacists can offer in this space [28]. It is also important to note that although the services offered by Australian community pharmacists in the area of pain may appear limited, there have been numerous pain intervention programmes developed as of recent. One example of this is the ‘PainWISE’ programme, an initiative in which community pharmacists undertake extensive additional training in both acute and chronic pain management and are perfectly placed to interact with patients and identify any issues that may warrant referral [34]. Another example of this is the ‘Chronic Pain MedsCheck Trial’ service, an initiative established in 2018 that is funded by the Australian Department of Health. The aim of this service is for pharmacists to engage with chronic pain patients and assess their pharmacological (as well as non-pharmacological) pain management strategies [35]. Although the Chronic Pain MedsCheck programme is in its early days, the outcomes of this service may address the insights raised in this study and may provide evidence to suggest that Australian community pharmacists are indeed capable of expanding on their pain management services that are attuned to meet the needs of their patients. Nevertheless, the importance of expanding and promoting the role of the pharmacist and community pharmacy services/programmes in the pain management space is clear.

### 4.4. Limitations and Future Directions

There were a number of limitations to this exploratory study. Although a broad range of demographics were captured, capturing a larger sample size would increase the generalisability of the results and therefore obtain a greater standpoint of the views and practices of Australian adults in pain management. Furthermore, although participants were asked to list all of their prescription analgesic medicines, this study did not specifically assess the frequency, dosage and history of prescription analgesic use. Additionally, a potential selection bias may exist that can influence the sample representativeness. Whilst there are many advantages to using questionnaires, they do have limitations, particularly in the area of assessing self-reported practice. Since this study included the use of a self-reported survey, response and/or recall bias is also possible. To minimise response bias, participants were reassured that their responses to the survey would remain completely anonymous. To extend this work, additional studies should be conducted to capture qualitative data and insights from the participants to further gauge their specific views and perceptions of community pharmacies/pharmacists in the pain management context. Furthermore, it would also be useful to conduct more studies assessing the effectiveness of tailored pharmacy-specific pain management services, for example longitudinal intervention studies implemented over a predetermined period of time in a community pharmacy setting. This would allow patients to provide insights into the pain management strategies they find beneficial, and thus allow Australian community pharmacists to expand on their current pain management professional services.

## 5. Conclusions

Approximately 20% of Australian adults live with chronic pain, and this number is set to rise with the aging population. It is a commonly presenting condition seen in the primary healthcare setting, and failure to provide adequate therapy almost always leads to negative clinical and economic outcomes. The results from this study confirm that the presence of chronic pain directly impacts the quality and functionality of life of sufferers. Research has found that inadequate treatment heightens the impact chronic pain has on the physical and psychological wellbeing of patients. It is therefore crucial that those who experience chronic pain are provided effective treatment in order to mitigate these health concerns. The predicament health professionals as well as pain sufferers face is the fact that adequately managing chronic pain is often a complex and long-term process. It is, therefore, no surprise that Australian community pharmacists frequently encounter chronic pain patients who require assistance. Community pharmacists play a pivotal role in the area of pain management; however, it appears that Australian adults are somewhat unaware of what community pharmacists can offer beyond the standard care of dispensing and counselling of prescription medicines. Furthermore, although most participants reported sound knowledge in their pain management, there is room for improvement. Thus, the results from this study suggest the need for community pharmacists to openly and further engage with patients experiencing pain with the aim of providing insights, education and awareness of the value they can add specifically in the context of pain management.

## Figures and Tables

**Table 1:** Demographic distributions from survey (*n* = 120). OTCs: Over-The-Counter medications.

Characteristic		*n* (%)
**Age**	18–24 years	14 (11.7)
	25–35 years	26 (21.7)
	36–45 years	22 (18.3)
	46–55 years	19 (15.8)
	56–65 years	20 (16.7)
	65+ years	19 (15.8)
**Gender**	Male	43 (35.8)
	Female	77 (64.2)
**Work Status**	Full-Time	33 (27.5)
	Part-Time	21 (17.5)
	Self-Employed	12 (10.0)
	Casual	8 (6.7)
	Unemployed due to pain	16 (13.3)
	Unemployed due to other reasons	6 (5.0)
	Student	4 (3.3)
	Retired	20 (16.7)
**Highest Qualification**	High School Certificate	24 (20.0)
	TAFE Diploma/Certificate	25 (20.8)
	University Degree	56 (46.7)
	Other	15 (12.5)
**Allergies/Sensitivities**	Yes	44 (36.7)
	No	76 (63.3)
**Comorbidities**	Yes	80 (66.7)
	No	40 (33.3)
**Regular use of prescription drugs (besides analgesics)**	Yes	80 (66.7)
	No	40 (33.3)
**Regular Use of OTCs (besides analgesics)**	Yes	54 (45.0)
	No	66 (55.0)
**Cigarette Smoking Status**	Do not smoke	98 (81.7)
	Occasional Smoker	8 (6.7)
	Regular Smoker	14 (11.7)
**Alcohol Consumption Status**	Do not drink	51 (42.8)
	Occasional Drinker	58 (48.3)
	Regular Drinker	11 (9.2)

**Table 2:** The nature of pain experienced by participants (*n* = 120).

Characteristic		*n* (%)
**Duration of Pain**	<1 month	11 (9.2)
	1–3 months	5 (4.2)
	3–6 months	4 (3.3)
	6–12 months	8 (6.7)
	>12 months	92 (76.7)
**Diagnosed with Chronic Pain by Physician**	Yes	77 (64.2)
	No	43 (35.8)
**Main Type of Pain**	Back Pain	46 (38.3)
	Musculoskeletal Pain	8 (6.7)
	Arthritis Pain	13 (10.8)
	Headache/Migraine Pain	8 (6.7)
	Neuropathic Pain	11 (9.2)
	Other ^1^	34 (28.3)
**Original Cause of Main Type of Pain**	Unknown	43 (35.8)
	Work-Related Injury	16 (13.3)
	Car Accident Related	6 (5.0)
	Sports Injury	19 (15.8)
	Other	36 (30.0)
**Allied Health Professionals consulted for pain relief**	Yes	83 (69.2)
	No	37 (30.8)
**Non-Pharmacological strategies used for pain relief**	Yes	91 (75.8)
	No	29 (24.2)

^1^ Other options mentioned by participants included: knee pain, shoulder tendonitis, foot/ankle pain, plantar fasciitis pain and rheumatoid arthritis pain.

**Table 3:** Pain scores and interference with quality/functionality of life (*n* = 120).

%	Percentage of Participants Selecting This Numerical Scale Score (Where 0 = No Pain/No Interference, 10 = Worst Pain/Complete Interference)										
	0	1	2	3	4	5	6	7	8	9	10
**Pain at its HIGHEST (in last week)**	3.3	0	1.7	3.3	4.2	6.7	15.0	17.5	26.7	15.8	5.8
**Pain at its LOWEST (in last week)**	20.0	10.8	15.8	17.5	17.5	7.5	5.8	2.5	1.7	0.8	0
**Pain on AVERAGE (in last week)**	1.7	3.3	5.8	10.0	16.7	21.7	15.0	13.3	10.8	1.7	0
**Pain RIGHT NOW**	13.3	7.5	12.5	10.0	7.5	12.5	12.5	11.7	9.2	3.3	0
**General Physical Activity**	5.8	1.7	4.2	5.8	5.8	9.2	14.2	15.8	15.8	5.0	16.7
**Mood**	9.2	0.8	5.0	10.0	9.2	10.8	11.7	16.7	10.0	9.2	7.5
**Getting out of bed**	11.7	5.8	10.0	8.3	8.3	11.7	8.3	8.3	13.3	3.3	10.8
**Movement**	11.7	3.3	8.3	10.8	5.8	14.2	10.0	13.3	10.0	4.2	8.3
**Walking Ability**	11.7	2.5	6.7	7.5	10.8	10.8	10.8	9.2	9.2	5.8	15.0
**Appetite**	29.2	11.7	6.7	13.3	7.5	8.3	6.7	5.8	3.3	2.5	5.0
**Sleep**	6.7	7.5	5.0	4.2	5.8	15.0	12.5	12.5	8.3	9.2	13.3
**Enjoyment of Life**	8.3	4.2	7.5	5.8	8.3	14.2	10.0	10.0	10.0	5.8	15.8

**Table 4:** Treatment options selected to treat pain symptoms of different severities (*n* = 120).

	*n* (%)		
Response	Mild Pain	Moderate Pain	Severe Pain
**Unsure**	11 (9.2)	4 (3.3)	8 (6.7)
**Nil**	68 (56.7)	14 (11.7)	1 (0.8)
**Paracetamol**	37 (30.8)	62 (51.7)	47 (39.2)
**Ibuprofen**	20 (16.7)	34 (28.3)	36 (30.0)
**Paracetamol + Ibuprofen combination**	7 (5.8)	13 (10.8)	16 (13.3)
**Aspirin**	4 (3.3)	9 (7.5)	9 (7.5)
**Diclofenac**	2 (1.7)	10 (8.3)	11 (9.2)
**Other Oral Anti-Inflammatories**	11 (9.2)	20 (16.7)	15 (12.5)
**Topical Anti-Inflammatories**	11 (9.2)	24 (20.0)	33 (27.5)
**Other Topical Analgesics**	8 (6.7)	19 (15.8)	21 (17.5)
**Visit Pharmacy/Pharmacist for help**	5 (4.2)	9 (7.5)	15 (12.5)
**Visit GP for help**	10 (8.3)	23 (19.2)	51 (42.5)
**Visit Hospital**	2 (1.7)	5 (4.2)	27 (22.5)
**Other ^1^**	18 (15.0)	38 (31.7)	49 (40.8)

^1^ Other options mentioned by participants included: non-pharmacological therapy only, such as massage and acupuncture, and the use of prescription-only drugs, such as triptans for migraine, a paracetamol/codeine combination and pregabalin for neuropathic pain.

**Table 5:** Participants’ views on pain management and Australian community pharmacies (*n* = 120).

Statement	*n* (%)				
	Strongly Disagree	Disagree	Neither	Agree	Strongly Agree
**I believe that I know how to manage my pain well.**	8 (6.7)	20 (16.7)	20 (16.7)	54 (45.0)	18 (15.0)
**I believe that the way I manage my pain could be improved.**	6 (5.0)	14 (11.7)	21 (17.5)	58 (48.3)	21 (17.5)
**I take my pain-relieving medicines regularly to help manage my pain.**	11 (9.2)	26 (21.7)	17 (14.2)	43 (35.8)	23 (19.2)
**I believe that my pain-relieving medicines are effective at managing my pain.**	6 (5.0)	22 (18.3)	25 (20.8)	54 (45.0)	13 (10.8)
**I find that visiting a pharmacy is helpful when it comes to managing my pain.**	14 (11.7)	20 (16.7)	43 (35.8)	28 (23.3)	15 (12.5)
**I find that speaking to the pharmacist is helpful when it comes to managing my pain.**	12 (10.0)	19 (15.8)	39 (32.5)	36 (30.0)	14 (11.7)
**I believe community pharmacies/pharmacists can improve their services and offer more in pain management.**	3 (2.5)	12 (10.0)	43 (35.8)	43 (35.8)	19 (15.8)
**If my pain-relieving medicines were available at supermarkets, I would rather go to a supermarket to purchase my pain-relievers than a pharmacy.**	36 (30.0)	40 (33.3)	20 (16.7)	15 (12.5)	9 (7.5)

## Supplementary Materials

The following are available online at https://www.mdpi.com/2226-4787/8/4/187/s1, File S1: Questionnaire—An exploration of the views and practices of people suffering from pain in the Australian community.
