# Supplementary material for: An Investigation of the Practices of Australian Adults Experiencing Pain and Their Views of Australian Community Pharmacy Pain Management Services
Source: Pharmacy (Basel). 2020 Oct 13;8(4):187. doi: 10.3390/pharmacy8040187 (PMC7711846; doi:10.3390/pharmacy8040187)
Supplement: Supplementary file 1 [file pharmacy-08-00187-s001.pdf]

Questionnaire – An exploration of the views and practices of people suffering from pain in the Australian community

PART 1

A few questions to build a picture of your health and background

1. Age?

☐ 18 – 24 years
☐ 25 – 35 years
☐ 36 – 45 years
☐ 46 – 55 years
☐ 56 – 65 years
☐ 65+ years

2. Gender?

☐ Male
☐ Female
☐ Do not identify as either Male or Female

3. What is your approximate weight? (in kg)

☐ My approximate weight is ______ kg

☐ I am unsure of my weight

4. What is your approximate height? (in cm)

☐ My approximate height is ______ cm

☐ I am unsure of my height

5. What is your current employment status?

☐ Employed Full Time
☐ Employed Part Time
☐ Self Employed
☐ Casual
☐ Unemployed due to pain
☐ Unemployed due to other reasons
☐ Student
☐ Retired

6. What is your highest education qualification?

☐ High School certificate
☐ TAFE diploma/certificate
☐ University degree
☐ Other

7. Besides your pain, do you have any other medical condition(s)? (Please tick all that applies)

☐ No, I do not have any other medical conditions
☐ High Blood Pressure
☐ Diabetes
☐ Asthma
☐ Arthritis
☐ Other/s (Please specify)

Insert your comments here:

8. Apart from pain-relieving medication(s), do you take any other regular prescription medicine(s)?

Yes ☐ No ☐

If so, please specify:

Insert your comments here:

9. Apart from pain-relieving medication(s), do you take any other regular 'over-the-counter' (i.e. non-prescription) medicine(s)?

Yes ☐ No ☐

If so, please specify:

Insert your comments here:

10. Do you have any allergies/sensitivities?

Yes ☐ No ☐

If so, please specify:

Insert your comments here:

11. Are you currently pregnant and/or breastfeeding?

☐ Neither/Not Applicable
☐ Pregnant
☐ Breastfeeding

12. What is your cigarette smoking status?

☐ I DO NOT smoke
☐ I OCCASIONALLY smoke (for example, I smoke cigarettes less than daily)
☐ I REGULARLY smoke (for example, I smoke cigarettes daily)

13. If you smoke cigarettes, do you smoke to help relieve your pain?

☐ Yes
☐ No
☐ I DO NOT smoke

14. What is your alcohol drinking status?

☐ I DO NOT drink
☐ I OCCASIONALLY drink alcohol (for example, not more than 1-2 standard drinks some days per week or less)
☐ I REGULARLY drink alcohol (for example, 1-2 standard drinks most days per week or more)

15. If you drink alcohol, do you drink to help relieve your pain?

☐ No
☐ Yes, I OCCASIONALLY drink alcohol to help relieve my pain
☐ Yes, I REGULARLY drink alcohol to help relieve my pain

PART 2

A few questions about your pain

16. How long have you been regularly experiencing pain symptoms for?

☐ Less than 1 month
☐ 1 to 3 months
☐ 3 to 6 months
☐ 6 to 12 months
☐ greater than 12 months

17. Have you been diagnosed with chronic pain by a health professional (e.g. Medical practitioner/GP)?

Yes ☐ No ☐

18. What is your MAIN type of pain you are experiencing? (Please select one)

- ☐ Back pain
- ☐ Tooth/Dental pain
- ☐ Muscle pain
- ☐ Arthritis pain
- ☐ Headache/Migraine pain
- ☐ Nerve pain
- ☐ Other (Please specify)

Insert your comments here:

19. What is the ORIGINAL CAUSE of your MAIN type of pain? (Please select one)

- ☐ I am UNSURE of what the cause of my pain is
- ☐ Work-related injury
- ☐ Car accident related injury
- ☐ Sports injury
- ☐ Other (Please specify)

Insert your comments here:

PART 3

A few questions about how you manage your pain

20. Please rate your pain by selecting one number that best describes the following: (0 = NO pain and 10 = WORST pain possible)

	NO PAIN 0	1	2	3	4	5	6	7	8	9	WORST PAIN 10
**Your pain at its HIGHEST in the last week**	☐	☐	☐	☐	☐	☐	☐	☐	☐	☐	☐
**Your pain at its LOWEST in the last week**	☐	☐	☐	☐	☐	☐	☐	☐	☐	☐	☐
**Your pain on AVERAGE**	☐	☐	☐	☐	☐	☐	☐	☐	☐	☐	☐
**Your pain RIGHT NOW**	☐	☐	☐	☐	☐	☐	☐	☐	☐	☐	☐

21. Please tick ALL of the pain-relieving medication(s) you are currently taking to help with your pain:

- ☐ I don't take any pain-reliving medications
- ☐ I cannot remember what pain-relieving medications I take
- ☐ Paracetamol tablets/capsules (e.g. Panadol/Panamax)
- ☐ Ibuprofen tablets/capsules (e.g. Nurofen)
- ☐ Paracetamol and Ibuprofen combination tablets (e.g. Maxigesic/Nuromol/Mersynofen)
- ☐ Aspirin tablets
- ☐ Diclofenac tablets (e.g. Voltaren)
- ☐ Other anti-inflammatories (e.g. Naproxen, Ponstan)
- ☐ Anti-inflammatory **skin gels/creams** (e.g. Voltaren Gel, Feldene Gel, Nurofen Gel)
- ☐ Other skin **gels/creams** (e.g. Deep Heat Gel, Ice Gel, Finalgon Cream, Tiger Balm, Zostrix/Capsaicin Cream)
- ☐ Other/s (Please specify)

Insert your comments here:

22. On a scale of 1-10 (with ONE being the LEAST effective and TEN being the MOST effective), how effective are your pain medications in relieving your pain?

LEAST EFFECTIVE									MOST EFFECTIVE
1	2	3	4	5	6	7	8	9	10
☐	☐	☐	☐	☐	☐	☐	☐	☐	☐

23. Please select any additional health professional(s) you see to help manage your pain other than your regular Doctor (Please tick all that applies)

- ☐ I don't see other health professionals besides my Doctor
- ☐ Physiotherapist
- ☐ Chiropractor
- ☐ Osteopath
- ☐ Occupational Therapist
- ☐ Psychologist
- ☐ Other (Please specify)

Insert your comments here:

24. **Do you do anything else to help manage your pain (e.g. exercise, hydrotherapy, heat packs, cold packs etc.)?**

Yes ☐ No ☐

If so, please specify:

Insert your comments here:

PART 4

A few specific questions relating to how you would manage your pain symptoms

25. **Please select ALL of the treatment options you would normally use when you experience MILD pain symptoms (that is, when your pain level is from 1-3 on the pain scale).**

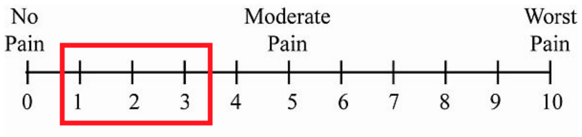

- ☐ I am not sure/don't know
- ☐ I wouldn't take any medicines for MILD pain symptoms
- ☐ Paracetamol tablets/capsules (e.g. Panadol/Panamax)
- ☐ Ibuprofen tablets/capsules (e.g. Nurofen)
- ☐ Paracetamol and Ibuprofen combination tablets (e.g. Maxigesic/Nuromol/Mersynofen)
- ☐ Aspirin tablets
- ☐ Diclofenac tablets (e.g. Voltaren)
- ☐ Other anti-inflammatories (e.g. Naproxen, Ponstan)
- ☐ Anti-inflammatory skin gels/creams (e.g. Voltaren Gel, Feldene Gel, Nurofen Gel)
- ☐ Other skin gels/creams (e.g. Deep Heat Gel, Ice Gel, Finalgon Cream, Tiger Balm, Zostrix/Capsaicin Cream)
- ☐ I would visit my pharmacy and speak to my pharmacist for help
- ☐ I would visit my GP for help
- ☐ I would go to the hospital
- ☐ Other/s (Please specify)

Insert your comments here:

26. Please select ALL of the treatment options you would normally use when you experience MODERATE pain symptoms (that is, when your pain level is from 4-7 on the pain scale).

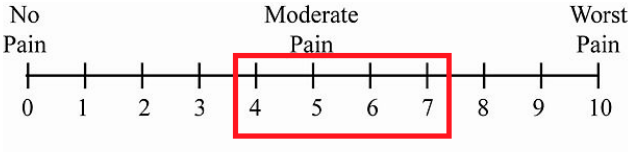

☐ I am not sure/don't know
☐ I wouldn't take any medicines for MODERATE pain symptoms
☐ Paracetamol tablets/capsules (e.g. Panadol/Panamax)
☐ Ibuprofen tablets/capsules (e.g. Nurofen)
☐ Paracetamol and Ibuprofen combination tablets (e.g. Maxigesic/Nuromol/Mersynofen)
☐ Aspirin tablets
☐ Diclofenac (e.g. Voltaren)
☐ Other anti-inflammatories (e.g. Naproxen, Ponstan)
☐ Anti-inflammatory skin gels/creams (e.g. Voltaren Gel, Feldene Gel, Nurofen Gel)
☐ Other skin gels/creams (e.g. Deep Heat Gel, Ice Gel, Finalgon Cream, Tiger Balm, Zostrix/Capsaicin Cream)
☐ I would visit my pharmacy and speak to my pharmacist for help
☐ I would visit my GP for help
☐ I would go to the hospital
☐ Other/s (Please specify)

Insert your comments here:

27. Please select ALL of the treatment options you would normally use when you experience SEVERE pain symptoms (that is, when your pain level is from 8-10 on the pain scale).

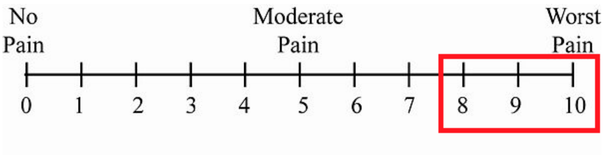

- ☐ I am not sure/don’t know
- ☐ I wouldn’t take any medicines for SEVERE pain symptoms
- ☐ Paracetamol tablets/capsules (e.g. Panadol/Panamax)
- ☐ Ibuprofen tablets/capsules (e.g. Nurofen)
- ☐ Paracetamol and Ibuprofen combination tablets (e.g. Maxigesic/Nuromol/Mersynofen)
- ☐ Aspirin tablets
- ☐ Diclofenac tablets (e.g. Voltaren)
- ☐ Other anti-inflammatories (e.g. Naproxen, Ponstan)
- ☐ Anti-inflammatory skin gels/creams (e.g. Voltaren Gel, Feldene Gel, Nurofen Gel)
- ☐ Other skin gels/creams (e.g. Deep Heat Gel, Ice Gel, Finalgon Cream, Tiger Balm, Zostrix/Capsaicin Cream)
- ☐ I would visit my pharmacy and speak to my pharmacist for help
- ☐ I would visit my GP for help
- ☐ I would go to the hospital
- ☐ Other/s (Please specify)

Insert your comments here:

PART 5

A few questions about your daily function

28. **Please tick the number that applies to each category.**

How does your pain interfere with the following?
(0 = Does not interfere and 10 = Completely interferes)

	Does not Interfere										Completely Interferes
	0	1	2	3	4	5	6	7	8	9	10
General Physical Activity	☐	☐	☐	☐	☐	☐	☐	☐	☐	☐	☐
Mood	☐	☐	☐	☐	☐	☐	☐	☐	☐	☐	☐
Getting out of bed	☐	☐	☐	☐	☐	☐	☐	☐	☐	☐	☐
Movement (e.g. getting around the house)	☐	☐	☐	☐	☐	☐	☐	☐	☐	☐	☐
Walking ability (e.g. walking to and from the shops)	☐	☐	☐	☐	☐	☐	☐	☐	☐	☐	☐
Appetite	☐	☐	☐	☐	☐	☐	☐	☐	☐	☐	☐
Sleep	☐	☐	☐	☐	☐	☐	☐	☐	☐	☐	☐
Enjoyment of life	☐	☐	☐	☐	☐	☐	☐	☐	☐	☐	☐

PART 6

Finally, a few questions about your views on pain management and Australian pharmacies

29. I believe that I know how to manage my pain well.

Strongly disagree ☐ Disagree ☐ Neither agree nor disagree ☐ Agree ☐ Strongly Agree ☐

30. I believe that the way I manage my pain could be improved.

Strongly disagree ☐ Disagree ☐ Neither agree nor disagree ☐ Agree ☐ Strongly Agree ☐

31. I take my pain-relieving medicines regularly to help manage my pain.

Strongly disagree ☐ Disagree ☐ Neither agree nor disagree ☐ Agree ☐ Strongly Agree ☐

32. I believe that my pain-relieving medicines are effective at managing my pain.

Strongly disagree ☐ Disagree ☐ Neither agree nor disagree ☐ Agree ☐ Strongly Agree ☐

33. I find that visiting a pharmacy is helpful when it comes to managing my pain.

Strongly disagree ☐ Disagree ☐ Neither agree nor disagree ☐ Agree ☐ Strongly Agree ☐

34. I find that speaking to the pharmacist is helpful when it comes to managing my pain.

Strongly disagree ☐ Disagree ☐ Neither agree nor disagree ☐ Agree ☐ Strongly Agree ☐

35. I believe that community pharmacies/pharmacists can improve their services and offer more in pain management.

Strongly disagree ☐ Disagree ☐ Neither agree nor disagree ☐ Agree ☐ Strongly Agree ☐

36. What would you like to see your community pharmacists do to improve their pain management services?

Insert your comments here:

37. If my pain relieving medicines were available at supermarkets, I would rather go to a supermarket to purchase my pain-relievers than a pharmacy.

Strongly disagree ☐ Disagree ☐ Neither agree nor disagree ☐ Agree ☐ Strongly Agree ☐

Why?

Insert your comments here:

Thank you for helping with this research project
